# Intrapulmonary lymph nodes

**DOI:** 10.36416/1806-3756/e20250278

**Published:** 2025-09-22

**Authors:** Edson Marchiori, Bruno Hochhegger, Gláucia Zanetti

**Affiliations:** 1. Universidade Federal do Rio de Janeiro, Rio de Janeiro (RJ) Brasil.; 2. University of Florida, Gainesville (FL) USA.

**Figure 1:**
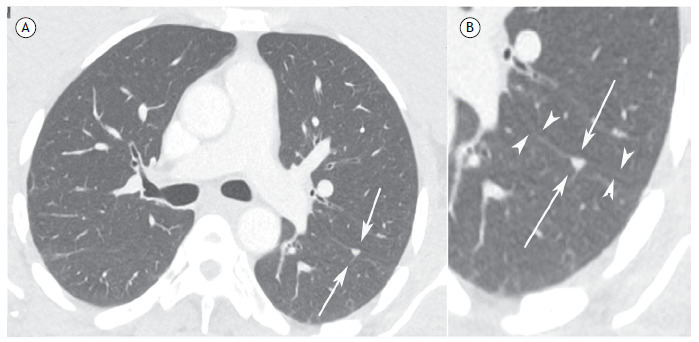
In A, axial CT image of the chest (lung window) showing a small triangular-shaped nodule in the posterior region of the left lung, closely related to the pleural fissure. In B, detail at higher magnification of the aforementioned nodule, clearly characterizing the triangular shape of the intrapulmonary lymph node (arrows) and its close relationship with the oblique fissure (arrowheads).

A 64-year-old man undergoing treatment for colon adenocarcinoma underwent imaging tests n for metastasis screening. A chest CT scan revealed a small, triangular nodule in the left lung, closely related to the oblique fissure (Figure 1).

Solitary pulmonary nodules (SPNs) remain a major diagnostic challenge for radiologists and pulmonologists. Recent technological advances in imaging techniques and the widespread use of CT have increased the frequency of pulmonary nodule detection. Small nodules (up to 5 mm in diameter) are commonly detected on CT images, and their clinical significance appears to differ significantly from that of larger nodules. However, this increased detection rate has not affected the basic issue of determining the nodule’s status-benign (no need for specific treatment) or indeterminate (potentially malignant). Most nodules are resected for diagnosis and definition of appropriate treatment.

Pulmonary lymph nodes are a common and underrecognized cause of SPN. These lymph nodes are usually found at the bifurcation of the bronchi, before the fourth branch, where they are called peribronchial lymph nodes. Occasionally, lymph nodes are present in the lung parenchyma, where they are called intrapulmonary lymph nodes (IPLN) or perifissural nodes. Differentiating IPLN from other small lung nodules on CT scans can be difficult, although clinically important. In particular, erroneous evaluation of an IPLN that is interpreted radiologically as a tumor nodule leads to overstaging and possible exclusion of surgical treatment in patients with primary lung cancer. Several CT features can aid in the differential diagnosis of IPLN. These lymph nodes can be oval, round, triangular, or trapezoidal, with well-defined borders, predominating in the subpleural regions of the lower lobes. They are frequently attached to the pleura or separated from the pleural surface by a few millimeters. IPLNs have thin, linear adhesions that extend from the nodule to the pleura. These linear densities have been shown to represent normal or thickened interlobular septa.^1^ ^-^ ^3^

It is important to emphasize that typical IPLNs, although generally benign, may show growth, without this indicating malignancy. Since they are lymph node-related, their growth may be due to reactive changes, especially inflammatory ones.^1^ ^-^ ^3^

In conclusion, IPLNs present imaging characteristics suggestive of benignity, which should be considered in the differential diagnosis of SPN. Correct identification of these lesions can reduce the number of unnecessary surgeries and follow-up examinations.
